# Effects of precipitation change and nitrogen addition on the composition, diversity, and molecular ecological network of soil bacterial communities in a desert steppe

**DOI:** 10.1371/journal.pone.0248194

**Published:** 2021-03-17

**Authors:** Meiqing Jia, Zhiwei Gao, Huijun Gu, Chenyu Zhao, Meiqi Liu, Fanhui Liu, Lina Xie, Lichun Wang, Guogang Zhang, Yuhua Liu, Guodong Han

**Affiliations:** 1 Key Laboratory of Water Resource and Environment, Tianjin Normal University, Tianjin, China; 2 College of Life Sciences, Tianjin Normal University, Tianjin, China; 3 Tianjin Key Laboratory of Animal and Plant Resistance, Tianjin Normal University, Tianjin, China; 4 Graduate School of Life and Environmental Sciences, University of Tsukuba, Tsukuba, Japan; 5 Institute of Agricultural Environment and Resource, Jilin Academy of Agricultural Sciences, Changchun, China; 6 Tianjin Agricultural Ecological Environment Monitoring and Agricultural Product Quality Testing Center, Tianjin, China; 7 College of Grassland, Resources and Environment, Inner Mongolia Agricultural University, Hohhot, China; Universidade de Coimbra, PORTUGAL

## Abstract

Currently, the impact of changes in precipitation and increased nitrogen(N) deposition on ecosystems has become a global problem. In this study, we conducted a 8-year field experiment to evaluate the effects of interaction between N deposition and precipitation change on soil bacterial communities in a desert steppe using high-throughput sequencing technology. The results revealed that soil bacterial communities were sensitive to precipitation addition but were highly tolerant to precipitation reduction. Reduced precipitation enhanced the competitive interactions of soil bacteria and made the ecological network more stable. Nitrogen addition weakened the effect of water addition in terms of soil bacterial diversity and community stability, and did not have an interactive influence. Moreover, decreased precipitation and increased N deposition did not have a superimposed effect on soil bacterial communities in the desert steppe. Soil pH, moisture content, and NH_4_^+^-N and total carbon were significantly related to the structure of bacterial communities in the desert steppe. Based on network analysis and relative abundance, we identified *Actinobacteria, Proteobacteria, Acidobacteria* and *Cyanobacteria* members as the most important keystone bacteria that responded to precipitation changes and N deposition in the soil of the desert steppe. In summary, we comprehensively analyzed the responses of the soil bacterial community to precipitation changes and N deposition in a desert steppe, which provides a model for studying the effects of ecological factors on bacterial communities worldwide.

## 1 Introduction

Change in precipitation and increase in N deposition are current global issues [1,2] that greatly affect the composition of terrestrial ecosystems. The global deposition of reactive N was 100 Tg N year^-1^ in 1995, which is predicted to increase to 200 Tg N year^-1^ by 2050 due to industrial pollution and agricultural practices [3,4]. Further, the effects of climate change are becoming obvious, especially the change in precipitation [5]. It is predicted that precipitation will increase in high latitudes and decrease in most subtropical regions [5]. In fact, compared with other grasslands, changes in precipitation have a greater impact on desert grassland ecosystems [6]. Compared with other steppe types, desert steppe is less stable and highly sensitive to disturbances due to climate change [7,8]. Water and N are key limiting factors of the survival of organisms inhabiting a desert steppe and are also two coupling factors in steppe ecosystems [9,10]. The dissolution of inorganic N is highly dependent on water [11–13]. Further, soil N addition enhances the photosynthetic capability of plants [14–16]. Therefore, understanding the responses of desert steppe ecosystems to changes in water and N content will play important roles in elucidating complex ecosystems and predicting the responses of ecosystems to global change.

Soil microbes are key components of below-ground ecosystems, as their diversity, composition, and activities are the major drivers of terrestrial ecosystem productivity and diversity [17–19]. Some studies have reported that precipitation and nitrogen deposition indirectly affect microbial communities by affecting plant communities [20,21]. At the same time, [22] pointed out that precipitation and nitrogen deposition directly affect the composition of microbial communities. Both soil chemistry and soil microbes were affected by N input and increased precipitation, especially when they were applied simultaneously [23,24]. For example, some studies show that changes in N addition and precipitation alter microbial communities through changes in soil pH [25]. The addition of nitrogen mainly affects the soil microbial community by lowering the pH of the soil, while increasing water promotes the increase of soil pH [26]. Similarly, precipitation affects microbial communities by increasing plant diversity, while the addition of nitrogen does the opposite [24]. In addition, N addition significantly decreases the relative abundance of soil fungi and increases the proportion of bacteria, whereas water addition has the opposite effects [22,27,28]. These results indicate that the response of the soil microbial community to N availability is highly dependent on changes in precipitation [29]. There are many studies on global change factors affecting the structure and function of microbial communities, some of them report on short term environmental changes from a few months to two years [30,31], and others report on the impact of singular climate factors on microbial communities [32]. However, there are few studies on microbial community responses to long-term precipitation changes and N deposition, especially in desert steppes [29]. Therefore, it is highly valuable to study the effects of long-term changes in water and N deposition on microbial communities in desert steppe. Moreover, the interaction between N deposition and precipitation change has practical significance in the responses of microbial ecosystems to complex global changes in multiple environmental factors.

Soil microbes do not typically live in isolation, but instead form complex inter-species networks that substantially regulate ecological community structure [33] and ecosystem function [34]. Ecological network analysis, a system analysis method based on random matrix theory, analyzes the interactions between different entities in a system [35]. It has recently been used to study complex microbial systems, such as ecosystem food web [36] and microbial community structure [34,37–39]. Analyzing the ecological network structure can reveal the relationship between ecosystem complexity and stability [40]. Complex interrelationships between species (such as predation, symbiosis, and competition) are important to the stability of the communities. Therefore, the complexity and stability of the ecological network established through the interrelationships of species can reflect the ability of the ecosystem to respond to changes in the external environment.

In this study, we conducted an 8-year field experiment to study the effects of interaction between N deposition and precipitation change on soil bacterial communities in a desert steppe using high-throughput sequencing technology. network analysis was an important method to examine the response of soil bacteria to changes in water and N. The objectives of this study were the followings: 1) Investigate whether soil bacterial communities are sensitive to long-term precipitation changes and N deposition, 2) explore the key species in the bacterial community affected by environmental factors, and 3) infer the impact of changes in precipitation and increased N deposition on desert steppe ecosystem.

## 2 Materials and methods

### 2.1 Study site

The experiment was conducted from June 24, 2006 to October 11, 2014 in a natural desert steppe ecosystem in Siziwang Banner (41 46′43.6"N, 111 53′41.7"E), Inner Mongolia, northern China. From 2006 to 2014, the mean soil moisture content was 5.42%, with the highest monthly soil moisture from June to September. The mean soil temperature was 6.44°C, with the highest monthly mean temperature in July (24.91°C). The average annual rainfall was 205.29 mm, and more than 75% of measured rainfall occurred from June to September (S1 Fig). The soil in the study site is Kastanozem (Haplic Calcisols according to the FAO classification) with a sandy loam texture., the basic soil properties at 0–30 cm depth, according to our background survey, are shown in S1 Table. The dominant plant species are *Stipa breviflora*, *Artemisia frigida* and *Cleistogenes Keng*.

### 2.2 Experimental design

**Fig 1:**
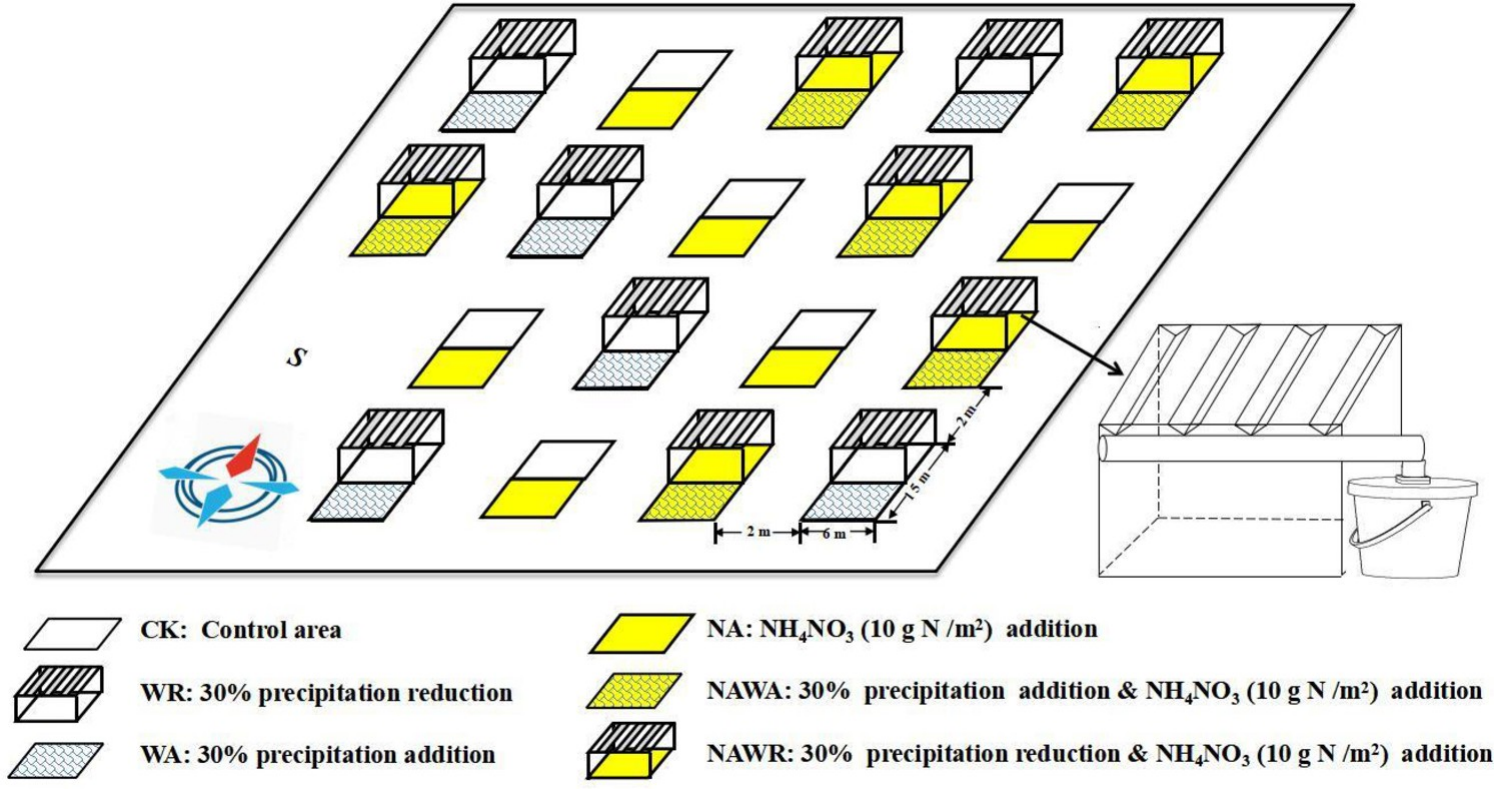
Experimental site and three-dimensional schematic diagram of experimental treatments. The experiment was conducted in a natural desert steppe ecosystem in Inner Mongolia. Eighteen 6 m × 15 m experimental plots were established in 2006. The following six treatments were randomly assigned: the control (CK), water addition (WA), water reduction (WR), N addition (NA), both N addition and water addition (NAWA), and both N addition and water reduction (NAWR).

Thirty-six 6 m × 15 m experimental plots were established in June 2006 (Fig 1). The following six treatments were randomly assigned: control (CK), water addition (WA), water reduction (WR), N addition (NA), simultaneous N addition and water addition (NAWA) and simultaneous N addition and water reduction treatments (NAWR). The distance between plots was approximately 2 m. A plastic baffle with a depth of one meter was inserted between each group of plots to prevent the exchange of moisture and nutrients between differently treated adjacent plots. An artificial drought shelter was used for water translocation. Twelve 6 m × 7.5 m artificial drought sheds were randomly constructed in the main plots, with a rain shelter of 100% transmittance on top of the shed. Each artificial drought shed eliminated 30% of natural precipitation in the WR treatment. The rainwater from the WR subplot is evenly sprayed to the WA subplot to supplement 30% of natural precipitation after each rainfall. In the NAWA and NAWR subplots, besides water regulation, N was applied once a year using granular NH_4_NO_3_ (10 g N m^-2^) [41] before rain in late April or early May. Each experimental treatment had six replicate plots. The experiment lasted 8 years, from 2006 to 2014. Rainfall and air temperature were obtained from meteorological stations established in the experimental site. A diagrammatic representation of the experimental device is shown in Fig 1. All necessary permits were obtained for the described study, which complied with all relevant regulations.

### 2.3 Soil sampling

Soil samples were collected in the middle of August in 2014, because the standing crop of the steppe communities reaches its annual peak from the middle to end of August. To obtain soil samples, 10 soil cores (30 cm depth and 5 cm diameter) were obtained from random locations in each plot, and then mixed to form one composite sample. Each soil core was divided into the following five layers: 0–2, 2–5, 5–10, 10–20 and 20–30 cm soil depths. After removing the roots, gravel and coarse fragments (2mm sterilized nylon mesh), soil samples were subdivided into two subsamples and stored at 4°C and -80°C for soil chemical analysis and DNA extraction, respectively.

### 2.4 Analysis of soil chemical parameters

The soil pH was measured using a pH electrode at a soil:water ratio of 1:2.5 (w/v) (Delta 320; Mettler-Toledo Instruments Co., Shanghai, China). Soil samples were dried in an oven at 105°C for 12 h and weighed to determine the soil moisture content. Soil nitrate (NO_3_^-^) and ammonium (NH_4_^+^) were extracted by shaking 5.0 g of fresh soil with 2 M KCl for 1 h, and their content was determined using a continuous flowing analyzer (SAN++, Skalar, Holland). The total C and total N concentrations of the soil were determined by dry combustion of duplicate subsamples using the LECO 2000 CHN Analyzer (LECO, Chicago, USA).

### 2.5 DNA extraction and high throughput sequencing

Total metagenomic DNA was isolated from soil samples using the Fast DNA SPIN Kit for Soil (MP Biotechnology, USA) according to manufacturer’s instructions. The quality of DNA was evaluated by agarose gel electrophoresis. The V4 region of 16S rRNA was amplified using specific primers (515F, 5’-GTGCCAGCMGCCGCG GTAA-3’ and 806R, 5’-GGACTACHVGGGTWTCTAAT-3’) with designated barcodes [42]. PCR reactions were carried out in 30 μL reactions with 15 μL of Phusion High-Fidelity PCR Master Mix (New England Biolabs); 1μl forward primer (10μM), 1μl reverse primer (10μM), and 10 ng template DNA. Thermal cycling consisted of initial denaturation at 98°C for 1 min, followed by 30 cycles of denaturation at 98°C for 10 s, annealing at 50°C for 30 s, elongation at 72°C for 30 s, and final 72°C for 5 min. Use TruSeq DNA PCR-Free Sample Preparation Kit for library construction. Libraries were assessed by the Qubit 2.0 Fluorometer (Thermo Scientific) and deep sequenced on the Miseq platform with PE300 strategy. The date has been uploaded to the National Microbiology Date Centers (NMDC10017737).

### 2.6 Bioinformatic analysis of 16S rRNA sequences

The raw readings are demultiplexed and assigned to the samples based on their unique barcodes. Trimmomatic (V0.35) was used for quality filtering with default parameters [43]. Then, high-quality paired-end reads were merged by using FLASH [44]. After removal of chimeric sequences based on the Silva database [45] and UChime algorithm [46], clean reads were subject to further analysis. Operational taxonomical units (OTUs) were clustered at a similarity threshold of 97% by using Uparse (v7.0.1001) [47]. All samples had sequencing depth of over 10 000 reads. Samples were normalized to 4269 sequences per sample. The Silva SSUrRNA (SSU128) Database was used based on the Mothur algorithm (thresholds: 0.8) to annotate taxonomic information for each representative sequence.

### 2.7 Network analysis

Network analysis is a method to determine the role of nodes in complex networks based on topological properties, and it offers new insights to determine key species and significant module members in microbe communities [48]. Network analysis was used to explore the co-occurrence patterns among the bacterial taxa. Analysis of ecological networks was performed using the Network Analyses Pipeline (http://ieg4.rccc.ou.edu/mena). More information on the pipeline, including its properties and relevant theories, can be found in [49–51]. Six co-occurrence networks of bacteria for the six treatments were built. For each network analysis, we used the data of samples from the five soil layers. The analysis was performed as follows. First, individual OTUs assigned into phylum level, was submitted to construct a network using the default settings. (OTUs appeared in more than half of all samples to be retained for subsequent analysis.) A similarity matrix was constructed based on Spearman’s Rho between pairwise OTUs. A cutoff value (similarity threshold, St = 0.6) for the similarity matrix was generated automatically using the default settings. A link was assigned between a pair of OTUs if the correlation between their abundance was larger than the St. Second, a set of measures (the number of nodes and links, average path length, number of positive and negative links, average connectivity, average clustering coefficient and modularity) was calculated to describe the topology of the resulting networks. The different roles of each node in the network were identified using the Zi plots (of values that measure within-module connectivity) and Pi plots (of values that measure among-module connectivity). These parameters can be used to develop Zi–Pi diagrams, which can be used to study key species in a community [49–51]. Third, Use the "igraph" and "psych" packages in the R (3.5.2) environment to calculate and draw network plots. Finally, the "randomize the network structure and then calculate network" was run. Random networks were generated for comparison with the topology of the empirical network; each link had the same probability of being assigned to any node [52].

### 2.8 Statistical analyses

The Shannon-Wiener diversity index is used to evaluate the diversity of different treatment methods [53]. Using "vegan" packaging in the R environment, redundant analysis is used to quantify the relative contribution of soil chemical factors to the composition of the bacterial community. The Monte Carlo displacement test was used to test the significance of soil chemical factors related to changes in bacterial communities. One-way analysis of variance was used to test the significance of the chemical properties of each soil layer under different treatment conditions.

The result of bacteria is expressed as the value of six replicate samples mixed into one sample (no replicate) by high-throughput sequencing. The results of soil physical and chemical properties are standardized average values of six replicate plots. Moreover, the statistical significance was accepted at P < 0.05. The software Sigmaplot 12.5 and SPSS were used for the statistical analyses.

### 2.9 Ethics statements

All experimental procedures in our research have been approved by the College of Grassland, Resources and Environment, Inner Mongolia Agricultural University. The sampling site was the temperature and nitrogen increase experiment plot in Siziwang Banner, College of Grassland, Resources and Environment, Inner Mongolia Agricultural University.

## 3 Results

### 3.1 Relationships between soil chemical properties and bacteria communities

Changes in the soil chemical properties among all the treatments in the five layers are listed in Table 1. Precipitation enrichment enhanced soil moisture in all the layers, especially in the 5–10, 10–20 and 20–30 cm layers, which reached a significant level (P < 0.05). Precipitation reduction decreased soil moisture in all the layers, but not significantly. However, precipitation enrichment and reduction had no significant effect on other chemical properties, including the soil pH, content of total C, TN, NH_4_^+^-N and NO_3_^—^N. At the same time, the changes of the total soil C and N concentrations were not significant under different treatments.

**Table 1:** Chemical properties of different soil layers under different treatments.

	Treat	pH	Moisture (%)	TC (%)	TN (%)	NO_3_^—^N (mg/kg)	NH_4_^—^N (mg/kg)
0–2 cm	CK	8.03 ± 0.12b	1.17 ± 0.16a	1.63 ± 0.08a	0.1775 ± 0.06a	4.70 ± 0.19a	2.35 ± 0.22a
	WA	7.87 ± 0.12b	1.48 ± 0.13a	1.78 ± 0.04a	0.1758 ± 0.03a	4.39 ± 0.14a	2.19 ± 0.13a
	WR	8.00 ± 0.16b	1.05 ± 0.07a	1.67 ± 0.05a	0.1775 ± 0.06a	4.45 ± 0.33a	2.22 ± 0.14a
	NA	7.37 ± 0.16a	1.27 ± 0.14a	1.65 ± 0.07a	0.1861 ± 0.05a	6.77 ± 0.39b	2.64 ± 0.14a
	NAWA	7.40 ± 0.37a	1.77 ± 0.04a	1.73 ± 0.10a	0.1783 ± 0.05a	6.84 ± 0.31b	2.79 ± 0.29a
	NAWR	7.35 ± 0.20a	1.09 ± 0.18a	1.64 ± 0.09a	0.1842 ± 0.02a	6.69 ± 0.41b	2.53 ± 0.18a
2–5 cm	CK	8.25 ± 0.03b	3.74 ± 0.10a	1.72 ± 0.07a	0.1825 ± 0.06a	4.86 ± 0.15ab	1.75 ± 0.18a
	WA	8.33 ± 0.21b	4.33 ± 0.32a	1.62 ± 0.08a	0.1842 ± 0.02a	4.55 ± 0.06a	1.69 ± 0.17a
	WR	8.31 ± 0.07b	3.33 ± 0.19a	1.67 ± 0.04a	0.1875 ± 0.06a	4.02 ± 0.24a	1.73 ± 0.07a
	NA	7.69 ± 0.17a	4.30 ± 0.13a	1.61 ± 0.09a	0.1871 ± 0.03a	5.47 ± 0.18b	2.41 ± 0.18a
	NAWA	7.74 ± 0.33a	4.32 ± 0.49a	1.68 ± 0.17a	0.1892 ± 0.08a	5.53 ± 0.47b	2.46 ± 0.03a
	NAWR	7.66 ± 0.24a	2.73 ± 0.23a	1.58 ± 0.09a	0.1850 ± 0.05a	5.37 ± 0.14b	2.38 ± 0.11a
5–10 cm	CK	8.52 ± 0.01a	5.84 ± 0.32ab	1.73 ± 0.08a	0.1850 ± 0.09a	3.43 ± 0.14a	1.71 ± 0.14a
	WA	8.56 ± 0.15a	6.31 ± 0.52b	1.54 ± 0.05a	0.1775 ± 0.04a	3.53 ± 0.18a	1.62 ± 0.15a
	WR	8.48 ± 0.11a	5.31 ± 0.08a	1.59 ± 0.05a	0.1775 ± 0.06a	3.24 ± 0.24a	1.73 ± 0.05a
	NA	8.19 ± 0.11a	5.91 ± 0.19ab	1.50 ± 0.02a	0.1811 ± 0.05a	5.17 ± 0.14b	2.27 ± 0.13a
	NAWA	8.33 ± 0.36a	7.34 ± 0.16b	1.52 ± 0.04a	0.1825 ± 0.03a	5.51 ± 0.07b	2.11 ± 0.09a
	NAWR	8.08 ± 0.14a	5.91 ± 0.14ab	1.50 ± 0.04a	0.1800 ± 0.03a	5.25 ± 0.17b	2.34 ± 0.18a
10–20 cm	CK	8.72 ± 0.01a	5.72 ± 0.41a	1.52 ± 0.05a	0.1625 ± 0.06a	3.52 ± 0.07a	1.89 ± 0.09a
	WA	8.76 ± 0.09a	6.95 ± 0.46b	1.37 ± 0.07a	0.1683 ± 0.05a	3.39 ± 0.10a	2.03 ± 0.09a
	WR	8.62 ± 0.07a	5.63 ± 0.15a	1.42 ± 0.11a	0.1600 ± 0.04a	3.02 ± 0.16a	2.02 ± 0.03a
	NA	8.44 ± 0.15a	5.69 ± 0.38a	1.32 ± 0.02a	0.1823 ± 0.02a	5.25 ± 0.20b	2.33 ± 0.16a
	NAWA	8.75 ± 0.14a	7.62 ± 0.50b	1.31 ± 0.14a	0.1692 ± 0.02a	5.38 ± 0.06b	2.38 ± 0.21a
	NAWR	8.26 ± 0.15a	5.67 ± 0.22a	1.33 ± 0.03a	0.1800 ± 0.02a	5.22 ± 0.29b	2.26 ± 0.17a
20–30 cm	CK	8.63 ± 0.07a	5.36 ± 0.42a	1.61 ± 0.19a	0.1425 ± 0.01a	3.04 ± 0.12a	2.04 ± 0.14a
	WA	8.83 ± 0.04a	7.14 ± 0.25b	1.46 ± 0.17a	0.1675 ± 0.03b	3.38 ± 0.18a	2.07 ± 0.13a
	WR	8.63 ± 0.06a	5.09 ± 0.27a	1.26 ± 0.10a	0.1450 ± 0.08a	3.18 ± 0.26a	1.91 ± 0.06a
	NA	8.44 ± 0.1 0a	5.69 ± 0.41a	1.29 ± 0.11a	0.1792 ± 0.04a	3.85 ± 0.03ab	2.47 ± 0.13a
	NAWA	8.75 ± 0.10a	7.05 ± 0.37b	1.34 ± 0.14a	0.1708 ± 0.03a	4.05 ± 0.34b	2.50 ± 0.14a
	NAWR	8.24 ± 0.1 0a	5.15 ± 0.34a	1.25 ± 0.11a	0.1742 ± 0.04a	3.81 ± 0.03ab	2.45 ± 0.13a

Note: Different letters in the same column indicate significant differences (P < 0.05) between treatments. WA represents the water addition treatment, WR represents the water reduction treatment, NA represents the N addition, NAWA represents the N addition and water addition treatment, and NAWR represents the N addition and water reduction treatment.

**Fig 2:**
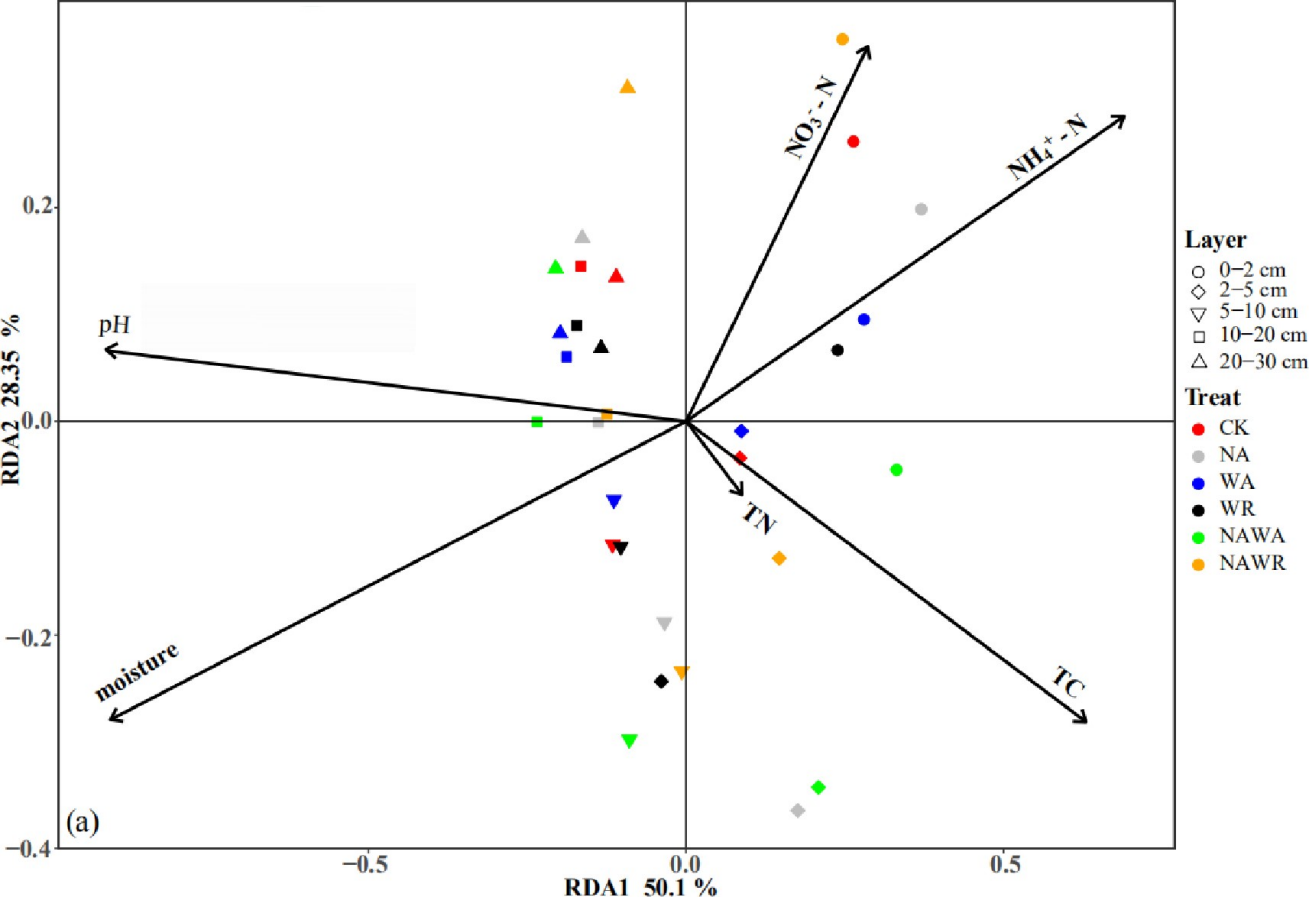
Redundancy analysis (RDA) of bacteria under different experimental treatments. (a) Species variables with environmental variables in different treatment soils and the percentage of variances explained by soil variables on the community structure of bacteria.

Redundancy analysis was performed to identify the relationship between environmental variables using soil physical and chemical properties and the soil bacterial community structures (Fig 2). The first two dimensions explained 50.1% and 28.35% of the total variances in the bacterial communities, respectively.

### 3.2 Diversity and composition of soil bacterial communities

**Fig 3:**
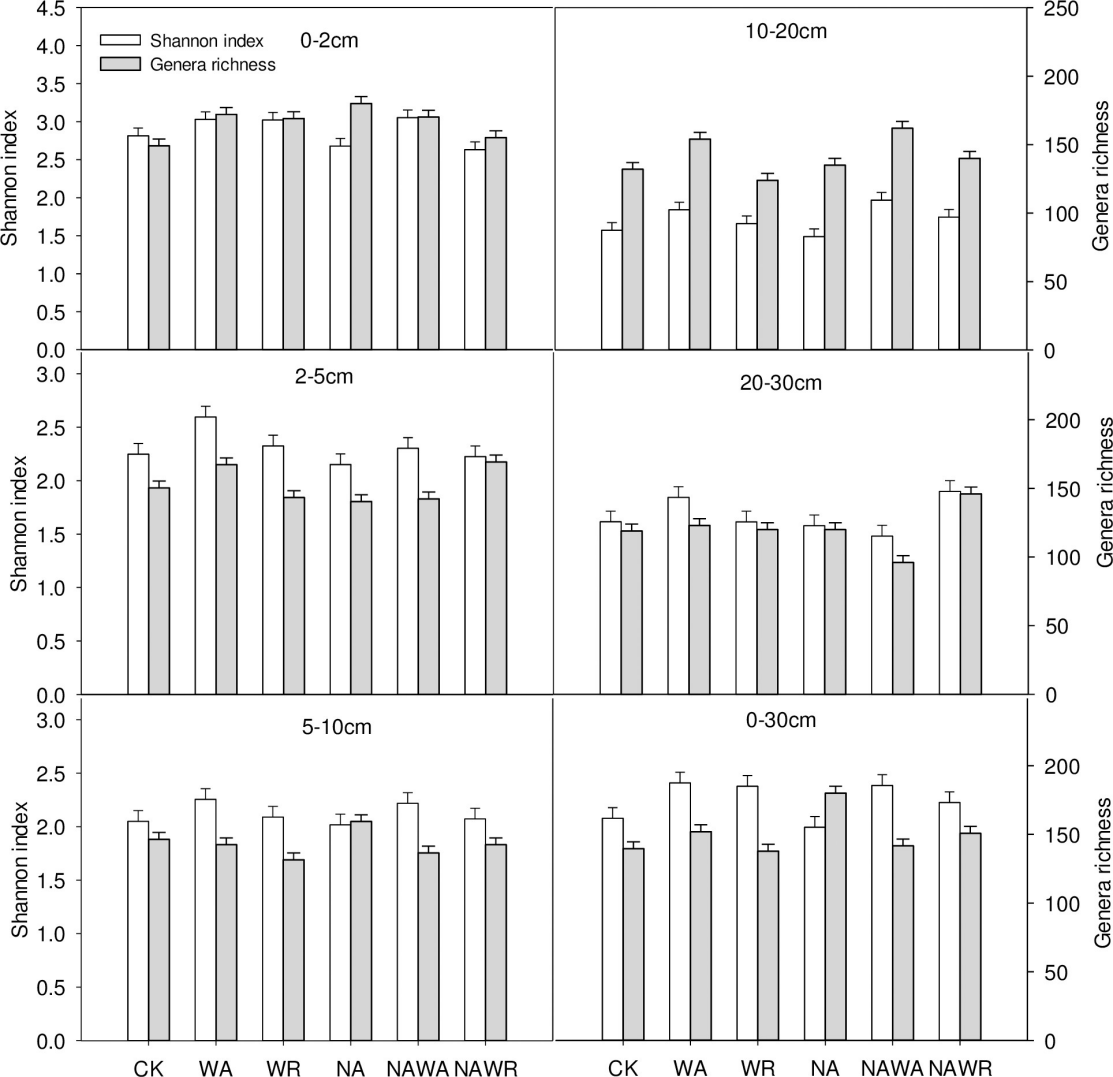
Diversity and genera richness of soil bacterial communities in five soil layers (a-e) and the entire 30-cm soil layer (f) under different experimental treatments. The experimental treatments included: the control (CK), water addition (WA), water reduction (WR), N addition (NA), both N addition and water addition (NAWA), and both N addition and water reduction (NAWR).

We obtained 287 286 high-quality soil bacterial 16S rRNA sequences. Bacterial sequences were affiliated with 30 phyla. Because the top 10 most abundant phyla contain the most community information (accounting for more than 95% of the community), this was used by us to describe the main components of soil bacterial communities. Shannon index and genera richness were used to evaluate the diversity of the soil bacterial community in desert steppe (Fig 3). Overall, compared with CK treatment, the soil bacterial community diversity of WA treatment increased, and the soil bacterial community of NA treatment decreased. The soil bacterial community diversity of WR treatment and CK treatment was the most similar, and there was no difference. Under the NAWA treatment, the diversity of the soil bacterial community increased compared with that under the CK. However, compared with that under the WA treatment, the bacterial community biodiversity under the NAWA treatment decreased. Moreover, the diversity of soil bacterial communities under the NAWR treatment did not decrease compared with that under the CK. That is, there was no superimposed effect of N deposition and drought on the diversity of soil bacterial communities in desert steppe.

**Fig 4:**
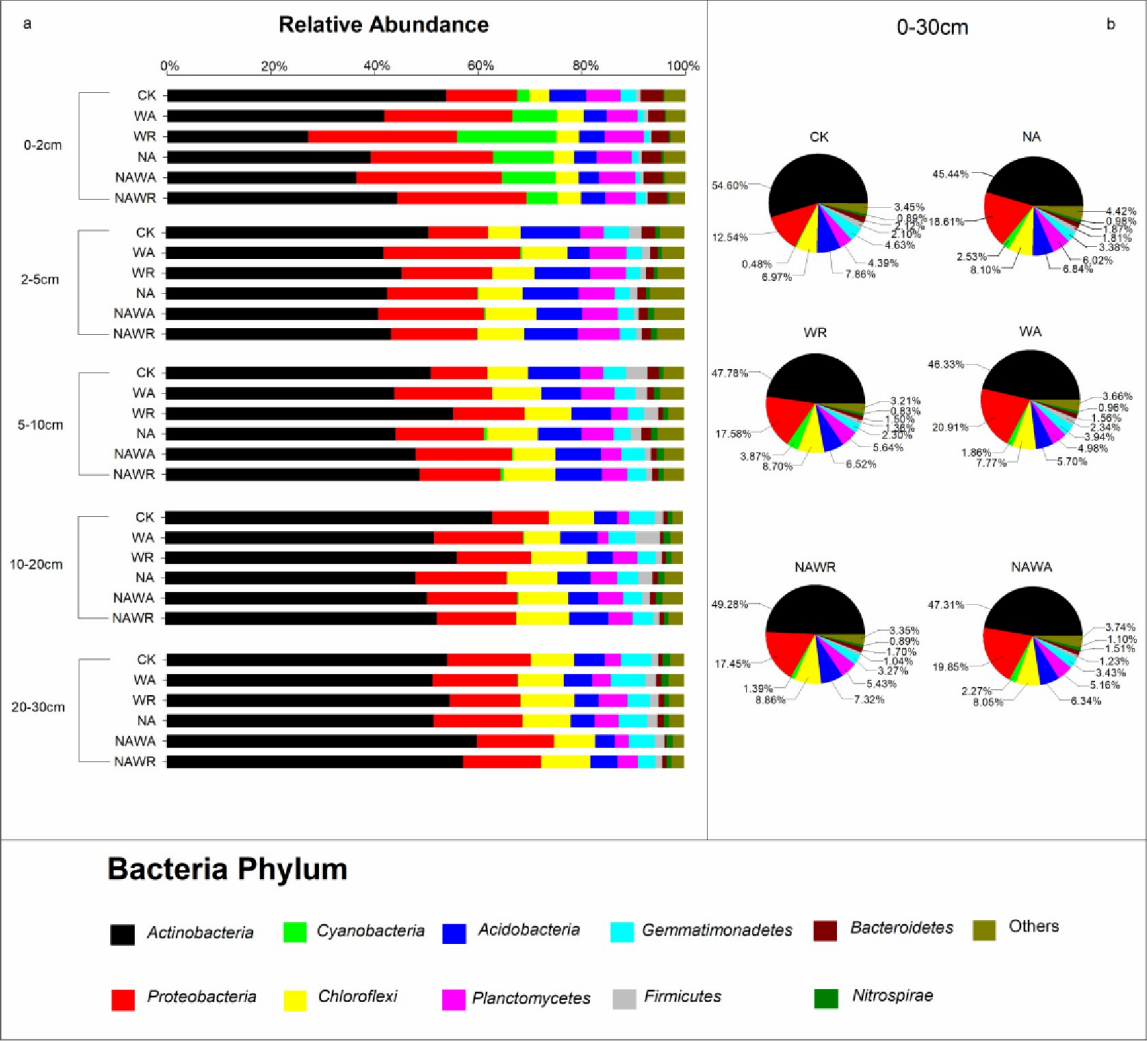
Bacterial community composition at the phylum level in different soil layers under different experimental treatments. (a) Relative abundance of the top 10 bacterial phyla and others in five soil layers: 0–2, 2–5, 5–10, 10–20 and 20–30 cm. (b) Relative abundance of the top 10 bacterial phyla and others in the entire 0–30-cm soil layer. The experimental treatments included the control (CK), water addition (WA), water reduction (WR), N addition (NA), both N addition and water addition (NAWA), and both N addition and water reduction (NAWR).

As shown in Fig 4, the differences in the relative abundance of all bacterial phyla of each treatment at the 0–2 cm soil layer was greater than that in other soil layers. In the 2–5 cm soil layer, the relative abundance of Acidobacteria was lower in all treatments compared to control. Especially, compared to other treatments, WR treatment caused highest relative decrease (26.62%). Compared with that under the CK, the relative abundance of Proteobacteria increased by 10.98% (WA), 14.96% (WR), 9.91% (NA), 14.33% (NAWA) and 11.15% (NAWR). The relative abundance of Cyanobacteria increased by 16.82% under the WR treatment compared with that under CK. We detected no differences in the relative abundance of any other bacterial phyla between the treatments.

In the 2–5 cm soil layer, the change in soil bacterial phyla was similar to that in the 0–2 cm soil layer. For example, the relative abundance of Proteobacteria increased by 14.78% under the WA treatment, but that of Acidobacteria decreased by 7.20% under the WR treatment, compared with that under the CK. In the 5–30 cm soil layer, the relative abundance of soil bacterial phyla was relatively stable except for that of Actinobacteria. Cyanobacteria appeared mainly in the 0–2 cm soil layer, and its relative abundance decreased gradually with increasing soil depth. In non-surface soil, the relative abundance of Cyanobacteria was higher than other treatments under NA and NAWA.

Overall, Actinobacteria, Proteobacteria and Chloroflexi were the dominant bacterial phyla in the 0–30 cm soil layer, accounting for 47.31%, 12.87% and 6.97% of all bacterial phyla, respectively. The relative abundance of Actinobacteria was stable among different treatments. Compared with that under the CK, the relative abundance of Proteobacteria increased by 8.37% (WA) and 7.31% (NAWA).

### 3.3 Network analysis of soil bacterial communities

**Fig 5:**
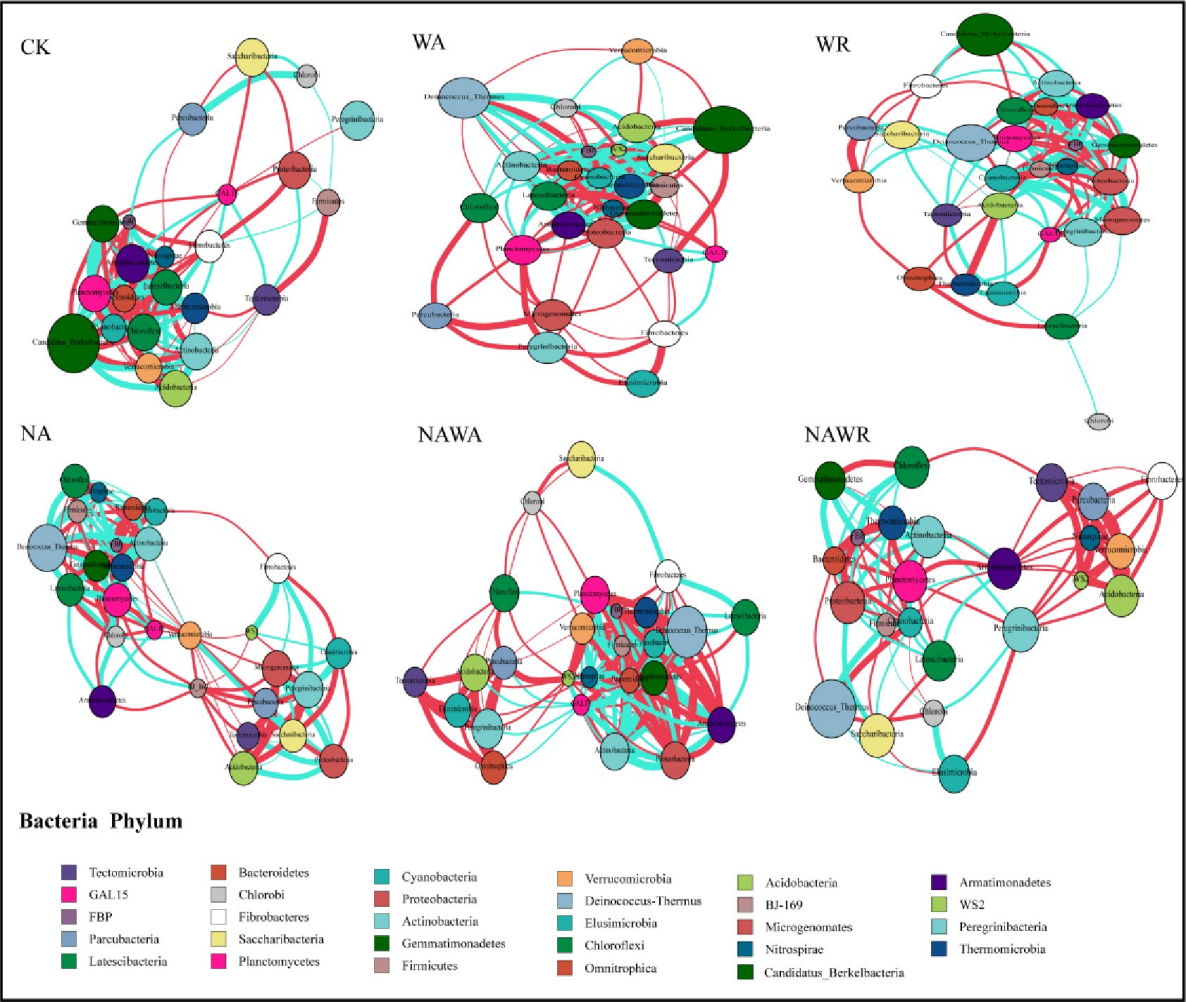
Ecological networks of bacterial communities under different experimental treatments. The experimental treatments included the control (CK), water addition (WA), water reduction (WR), N addition (NA), both N addition and water addition (NAWA), and both N addition and water reduction (NAWR). Network plots were drawn using using the “igraph” and “psych” package in the R environment. The node colors represent bacterial phyla of the operational taxonomic units (OTUs). The node size is proportional to the square root of the abundance of the OTU. The red edges indicate positive relationships and green edges indicate negative relationships. The thickness of the connection between two phyla represents the size of the correlation coefficient. (All depths of the same treatment are included).

The visualized networks of soil bacterial communities showed the roles of different phyla and their interactions with the other phyla under different treatments (Fig 5). There were obvious differences among the six empirical networks of the bacterial communities under the CK, NA, WA, WR, NAWA and NAWR treatments and their corresponding random networks in terms of the average path distance, average clustering coefficient, and average modularity (Table 2). Compared with the CK treatment, the topological structure of the soil bacterial network under different treatments showed greater differences, corresponding to differences in the major characteristics of bacterial communities (Fig 5, Table 2). The total number of links in the WA network increased by 11.76% (Table 2) compared with that in the CK network, whereas there were no obvious changes in the average connectivity. The total number of links and average connectivity in the NAWA treatment network increased by 7.19% and 1.33 compared with those in the CK network, respectively. In the WR treatment network, these values decreased by 9.15% and 3.76, respectively, and in the NAWR treatment network, these values decreased by 14.05% and 3.95, respectively. The percentage of negative links in the WA, NAWA and NAWR networks decreased, whereas that in the WR network increased. The average path distance of WA and NAWA networks decreased, while the average path distance of WR and NAWR networks increased. Compared with the CK network, the modularity of these five networks have been improved.

**Table 2:** Major properties of bacterial networks under different experimental treatments and the associated random networks.

	CK	WA	WR	NA	NAWA	NAWR
**Empirical networks**						
Total nodes	27	29	29	28	27	29
Total links	306	342	278	324	328	263
Negative links	44.44%	40.94%	47.48%	40.43%	37.19%	31.18%
Positive links	55.56%	59.06%	52.52%	59.57%	62.81%	68.82%
Avg. connectivity	22.5185	22.2069	18.7586	21.9310	23.8519	18.5714
Avg. path distance	1.7037	1.6921	1.8325	1.8498	1.6296	1.8201
Avg. clustering coefficient	0.7234	0.6732	0.6297	0.7352	0.7622	0.6220
Modularity	0.1529	0.1646	0.2050	0.3708	0.2616	0.3289
**Random networks**						
Avg. path distance	1.5307	1.5709	1.6426	1.5759	1.5046	1.6307
Avg. clustering coefficient	0.7202	0.6754	0.6011	0.6680	0.7423	0.6133
Modularity	0.0392	0.0508	0.0715	0.0480	0.0258	0.0731

Note: CK represents the control, WA represents the water addition treatment, WR represents the water reduction treatment, NA represents the N addition, NAWA represents the N addition and water addition treatment, and NAWR represents the N addition and water reduction treatment.

**Fig 6:**
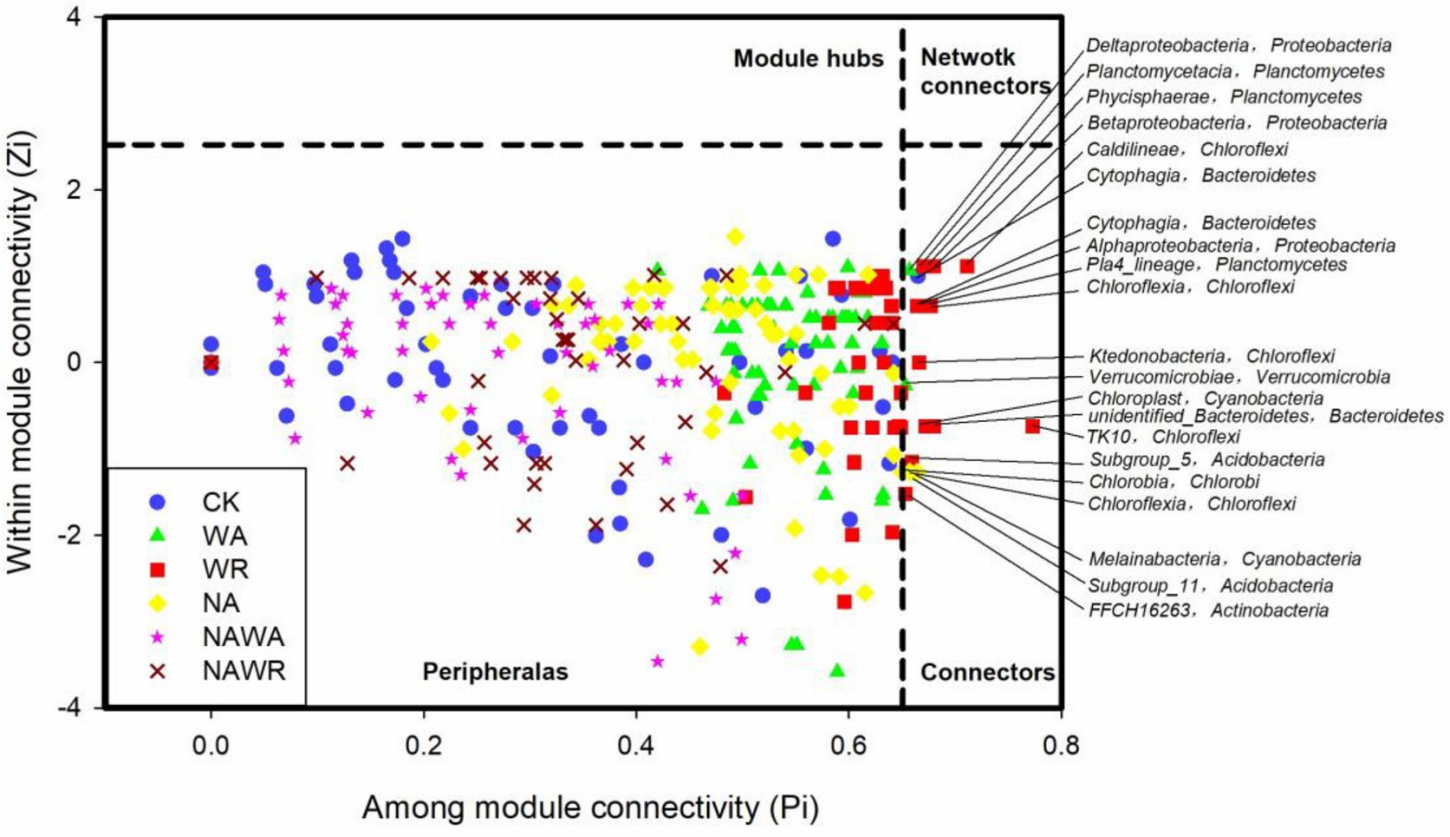
Zi–Pi plot showing distribution of bacterial classes based on their topological roles in bacterial networks. The experimental treatments included the control (CK), water addition (WA), water reduction (WR), N addition (NA), both N addition and water addition (NAWA), and both N addition and water reduction (NAWR). Each symbol represents a class under different experimental treatments. According to the classifications, all nodes are divided into the following four categories distributed in four regions: Z ≥ 2.5 and P ≤ 0.62 indicate module hubs (regarded as generalists); Z ≥ 2.5 and P > 0.62 suggest network connectors (regarded as supergeneralists); Z < 2.5 and P ≤ 0.62 show peripherals (regarded as specialists); and Z < 2.5 and P > 0.62 indicate connectors (regarded as generalists).

The Zi–Pi plot in Fig 6 showed the distribution of bacteria based on their topological roles in soil bacterial networks. As shown in Fig 6, the majority of the nodes (> 95%) under different treatment networks were categorized as peripherals (specialists, Zi < 2.5 and Pi < 0.62, indicating nodes with only a few links that are predominant to other nodes within their modules). 21 nodes were classified as connectors (generalists, Zi < 2.5 and Pi ≥ 0.62, which are predominantly linked to several modules). Fifteen of these 21 nodes belonged to Planctomycetes (3 OTU), Proteobacteria (2 OTU), Bacteroidetes (2 OTU), Chloroflexi (4 OTU), Acidobacteria (2 OTU), Actinobacteria (1 OTU) and Cyanobacteria (1 OTU) from the WR treatment network; two belonged to Proteobacteria (1 OTU) and Verrucomicrobia (1 OTU) from the WA treatment network; three belonged to Chlorobi (1 OTU), Chloroflexi (1 OTU) and Cyanobacteria (1 OTU) from the NA treatment network and one belonged to Bacteroidetes (1 OTU) from the CK treatment network. Our results suggest that there was no node in the network hub (super generalists, Zi ≥ 2.5 and Pi ≥ 0.62) category that existed as both a module hub and connector. As expected, more connectors (generalists) existed in the water regulation networks than in the CK network.

## 4 Discussion

In the 10-year experiment, we studied the effects of precipitation changes and N deposition on the soil bacterial community in the desert steppe environment. The results showed that precipitation changes and N deposition not only potentially affect the composition and diversity of soil bacterial communities, but also change their ecological network, and thus affect the stability of soil bacterial community. This indicates that the obscure mechanisms underlying microbial responses to global change should be analyzed.

### 4.1 Effects of precipitation changes on the soil bacterial community

Our study area is located in arid and semi-arid steppes where the water content is a major limiting factor of steppe productivity. We expected that drought simulation for 8 consecutive years would significantly reduce the diversity of soil microbial communities, but our results were contrary to our expectations. Our results indicated that the diversity of the soil bacterial community was sensitive to increased precipitation, but they showed a strong tolerance to drought. This might be because soil bacteria inhabiting the arid environment long term have a great potential for drought tolerance. The distribution of precipitation throughout the year in our experimental area was uneven. More than 75% of the measured rainfall occurred from June to September (S1 Fig). Long-term and frequent drying-wetting cycle stress might be one of the reasons that the microbial community has great potential for drought tolerance [32]. Compared to that in other soils, [32] pointed out that microbes had a greater ability to maintain their function in response to repeated water stress in grassland soil. In addition, some research shows that a change in precipitation has no significant effect on the composition of the bacterial community of the top soil or rhizosphere soil, but does affect the abundance of bacteria [23,54]. This is consistent with our results. Precipitation changes did not significantly affect the composition of the soil bacterial community at the phylum level, but only affected a few species sensitive to soil moisture.

Precipitation change also affected the stability of the soil bacterial community in the desert steppe. The co-occurrence of bacteria within the community leads to the complexity of the community structure and maintains the stability of the community [30,37,55]. In terms of community stability and sensitivity, increased modularity reduced the influence of environmental factors on the entire network and enhanced the stability of the soil bacterial community. The shorter average path distance indicated higher sensitivity and more rapid response to water addition. Increased modularity suggested that water reduction reduced the influence of environmental factors on the entire network and enhanced the stability of the soil bacterial community. The longer average path distance indicated a lower sensitivity and slower response to water reduction. This might be related to the adaptation of bacterial communities to the drought conditions of desert steppe.

### 4.2 Effects of water on the soil bacterial communities with N enrichment

Water and N addition increased the influence of environmental factors on the entire network and weakened the stability of the soil bacterial community, whereas N addition and water reduction had opposite effects on the stability and sensitivity of the soil bacterial community. Actually, the effect of water and N on soil microbial communities in desert steppe has been reported previously. Currently, there are two opinions about the effect of water and N addition on the soil bacterial communities in desert steppe. First, water and N addition might interactively affect soil microbial communities in semiarid steppe [56]. Second, water and N addition can have opposite effects on soil bacterial communities [13,27,57]. Our study indicated that N addition weakened the effect of water addition in terms of bacterial diversity and community stability, and they did not generate interactive influence. Moreover, N addition and water reduction together did not affect the diversity and stability of soil bacterial communities; that is, N deposition and drought did not have a cumulative effect on soil bacterial communities in desert steppe. This complexity might be related to the efficiency of water and N use in soil. Although precipitation enrichment could compensate the N requirement for microbe growth in arid soil by promoting N mineralization, increasing water availability induces substrate loss through nitrate leaching [58]. It has been demonstrated that precipitation conditions could lead to nutrients loss, thereby inducing soil nutrient deficiency [59]. These results indicated that precipitation plays an essential role in nutrient budget, which in turn influences microbial functional groups. In addition, precipitation regimes could alleviate the negative effects of soil pH induced by N addition on soil microbial communities [60]. Thus, the absence of interactive effects confirmed that the response of the soil microbial community to N addition could be mediated by precipitation management in desert steppe.

Moreover, N addition has a negligible effect on soil bacterial communities, which can also be explained by the limitation in soil C. As reported previously, N addition can also lead to a decrease in soil microbial community biomass and activity in systems that are C limited [61]. Total soil N and C concentrations were not significantly influenced by N addition in our field experiment site. A previous study has shown that N addition caused an increase in aboveground net primary productivity, but root biomass did not proportionally increase with aboveground biomass in desert steppe [62]. This can explain why the total soil C concentration did not increase consistently in plots with N addition. Heterotrophic microbes are thought to be C limited when the C:N ratio is below 30, and N limited when the C:N ratio is above 30 [63]. The C:N ratio in our study ranged from 7.35 to 9.98. At the same time, the results of redundancy analysis (RDA) (Fig 2B) also showed that total organic carbon was significantly correlated with the bacterial community structure (P < 0.05). These results suggest that soil microbes in our study were C limited, which might greatly influence the soil microbe community in desert steppe.

### 4.3 Responses of the keystone bacteria to precipitation changes and N deposition

Generalists are considered to be key organisms in a community [50]. In this study, 21 nodes from the WR, WA and CK treatments were categorized as connectors (generalists), which were highly connected to several modules. The 21 generalists represented nine phyla, *viz*. Planctomycetes, Proteobacteria, Bacteroidetes, Chloroflexi, Acidobacteria, Actinobacteria, Chlorobi, Cyanobacteria and Verrucomicrobia, which can be the key phyla affecting the stability of soil microbial communities in desert steppe.

At the same time, the difference in the relative abundance of bacteria in the community composition is also an indicator to determine the keystone bacteria. We found that there are seven bacterial phyla that account for more than 90% of the total community, namely Actinobacteria, Proteobacteria, Cyanobacteria, Chloroflexi, Acidobacteria, Planctomycetes and Gemmatimonadetes. Among them, the relative abundance of Actinobacteria, Proteobacteria, Acidobacteria and Cyanobacteria greatly differed with precipitation changes and N deposition treatments. In addition, the intersection of the key phyla that affect the ecological network and community composition may be the most important keystone bacteria responding to changes in precipitation and N deposition in desert desert steppe. In this study, we found that these four phyla are the keystone phyla of the ecological network, and the relative abundance of their bacterial communities will change with precipitation and N deposition treatments. Therefore, Actinobacteria, Proteobacteria, Acidobacteria and Cyanobacteria are the key phyla affecting the composition of soil microbial communities in desert steppe.

In fact, there are still many deficiencies in our research on network analysis. The effects of water-nitrogen on the stability and sensitivity of the ecological network can help us understand the overall response of soil bacterial communities to environmental changes. At the same time, keystone species that respond significantly to environmental changes can be identified. Although we can identify keystone phyla by the method of network analysis, we still cannot recognize sensitive species and conservative species in keystone phyla. We still think it is a practical approach to narrow down the range of key species. The key species we identified need to be further validated by systematic experiments.

## 5 Conclusions

The response of the composition, diversity, and network of soil bacterial communities to precipitation change and N deposition exhibited a certain degree of uniqueness in desert steppe. Decreasing precipitation and increasing N deposition did not exhibit a cumulative effect on soil bacterial communities in the desert steppe. This means that even if the diversity and functions of the ecosystem are expected to be affected by N deposition in the future, combination with precipitation may help maintain the stability of the semi-arid steppe ecosystem under climate change. Based on network analysis and relative abundance, we identified Actinobacteria, Proteobacteria, Acidobacteria and Cyanobacteria as the most important keystone bacteria responding to precipitation changes and N deposition in the soil of desert steppe. In future research, we should focus on the underlying mechanisms of these key phyla to climate change and find the key species which are sensitive and conservative to global change in the keystone phyla.

## Supporting information

S1 Fig Monthly precipitation from 2011 to 2016 in the experimental site. (TIF)

S1 Table Background nutrients content of upper 30 cm. (DOCX)
