# Supplementary figures and images for: Effects of precipitation change and nitrogen addition on the composition, diversity, and molecular ecological network of soil bacterial communities in a desert steppe
Source: PLoS One. 2021 Mar 17;16(3):e0248194. doi: 10.1371/journal.pone.0248194 (PMC7968660; doi:10.1371/journal.pone.0248194)

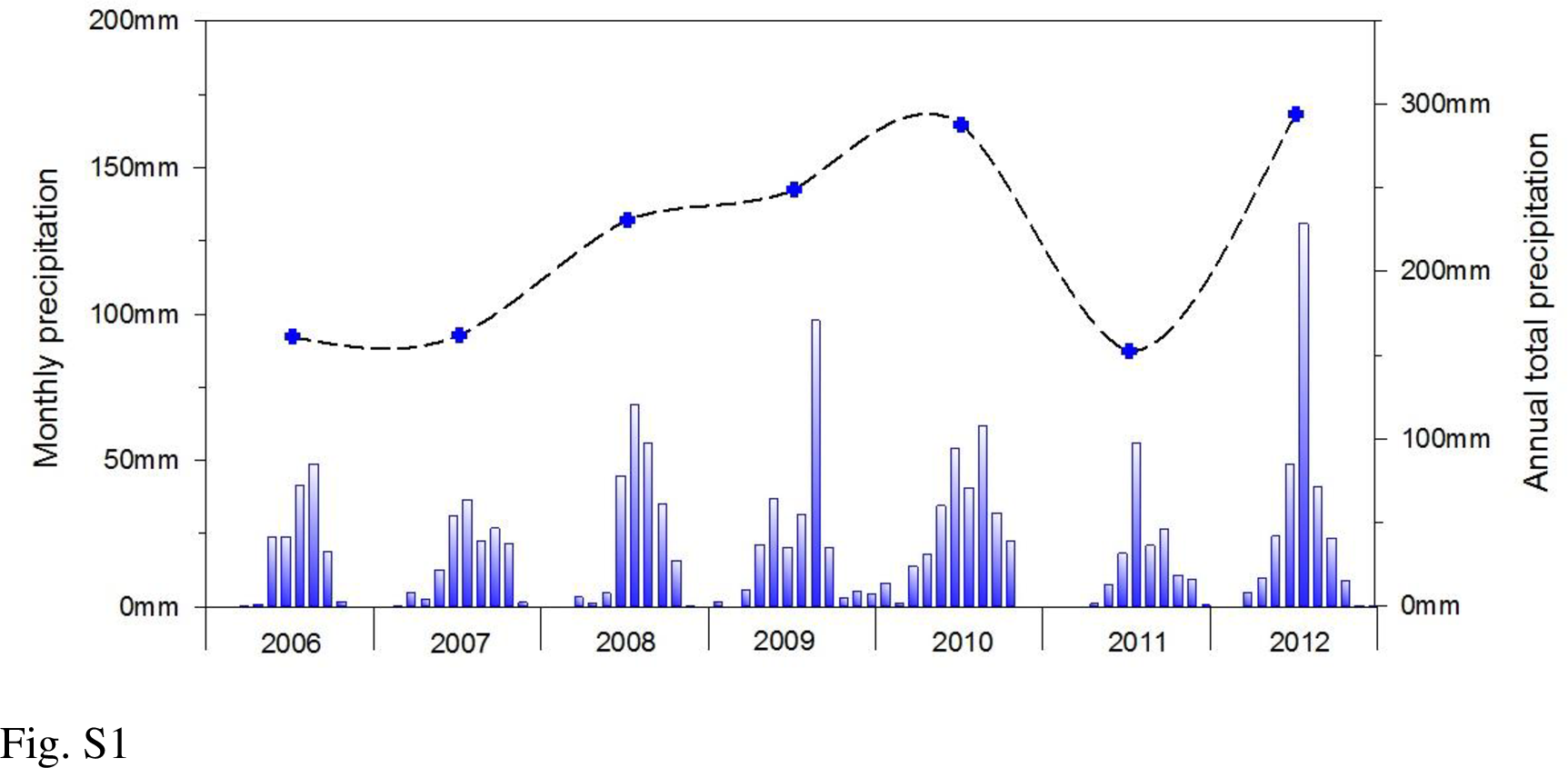

Supplement: S1 Fig — (TIF) [file pone.0248194.s001.tif]
