# Supplementary material for: Effects of precipitation change and nitrogen addition on the composition, diversity, and molecular ecological network of soil bacterial communities in a desert steppe
Source: PLoS One. 2021 Mar 17;16(3):e0248194. doi: 10.1371/journal.pone.0248194 (PMC7968660; doi:10.1371/journal.pone.0248194)
Supplement: S1 Table — (DOCX) [file pone.0248194.s002.docx]

S1 Table. background nutrients content of upper 30 cm.

| Total N content (g/kg) | Total P content (g/kg) | Total K content (g/kg) | Available N content (mg/kg) | Available P content (mg/kg) | Available K content (mg/kg) |
| --- | --- | --- | --- | --- | --- |
| 1.27 | 0.31 | 34.45 | 44.07 | 8.60 | 146.27 |
